# Surgical management of spinal metastases from primary lung carcinoma: demographics, clinical characteristics, and outcomes—A retrospective analysis

**DOI:** 10.3389/fsurg.2026.1743422

**Published:** 2026-02-26

**Authors:** Jun Li, Yuhan Zheng, Xiaohua Lv, Rong Zeng, Yating Zhao, Yucheng Xiang, Ke Zhan, Congcong Liu, Houqing Long, Ke Chen

**Affiliations:** 1 Department of Spine Surgery, Shenzhen People's Hospital (The First Affiliated Hospital, Southern University of Science and Technology; The Second Clinical Medical College, Jinan University), Shenzhen, China; 2 Department of Vascular Surgery, Shenzhen Luohu Hospital Group Luohu People's Hospital, Shenzhen, China; 3 School of Pharmacy, Guangdong Medical University, Dongguan, China; 4 Department of Pain Medicine, Shenzhen People's Hospital (The First Affiliated Hospital, Southern University of Science and Technology; The Second Clinical Medical College, Jinan University), Shenzhen, China

**Keywords:** lung cancer, prognosis, spinal metastasis, surgical treatment, survival analysis

## Abstract

**Objective:**

This study aims to evaluate the efficacy of palliative surgery in patients with spinal metastases from lung cancer and to identify prognostic factors affecting postoperative 1-year survival, providing clinical treatment references for these patients.

**Methods:**

Clinical data of 55 patients with spinal metastases from lung cancer who underwent surgery at Shenzhen People's Hospital from January 2017 to December 2022 were analyzed. Improvements in preoperative and postoperative visual pain scores, ODI scores, and Frankel grades were assessed. Kaplan–Meier method was used to plot survival curves, and the Cox proportional hazards model was employed to analyze various factors influencing postoperative 1-year survival.

**Results:**

Surgical treatment helped alleviate pain, maintain or improve neurological function, and enhance the quality of life. Among the 55 patients, 23 (41.82%) died, and 32 (58.18%) survived, with a median survival time of 16.83 months (95% CI: 9.88, 23.78) and a one-year survival rate of 58.18%.Factors influencing postoperative 1-year survival included ODI score one-month post-surgery, presence of visceral metastases, and postoperative bone modifying agents (BMA). Multivariate Cox proportional hazards model survival analysis indicated that the presence of visceral metastases and postoperative BMA were independent factors affecting one-year survival in these patients.

**Conclusion:**

Surgical treatment effectively alleviates pain, maintains or improves neurological function, and enhances the quality of life in patients with spinal metastases from lung cancer. The presence of visceral metastases and postoperative BMA are independent factors influencing postoperative 1-year survival.

## Highlights

- Surgical treatment effectively alleviates pain, maintains or improves neurological function, and enhances the quality of life in patients with spinal metastases from lung cancer.
- The presence of visceral metastases and postoperative BMA are independent factors influencing postoperative 1-year survival.

## 1 Introduction

Malignant tumors have emerged as a major global public health challenge. Accumulating evidence from the latest epidemiological data indicates a persistent upward trend in the incidence of malignant tumors (1, 2). Spinal metastasis occurs in approximately 30%–70% of patients with malignant tumors (3–6). With advances in multimodal treatment for malignant tumors, the survival duration of patients with malignant tumors has been prolonged even in the setting of multiple metastases. Clinical manifestations of spinal metastasis may include pain, severe weakness secondary to tumor compression of the spinal cord or nerve roots, or pathological fractures, which can lead to acute deterioration of the patient's condition and significantly impaired quality of life (3, 5, 7–10).

Recent studies have demonstrated that lung cancer is the leading cause of cancer incidence and mortality in China (1, 3, 11). Lung cancer is characterized by high invasiveness, poor prognosis, and short survival expectancy. It is estimated that 64.3%–64.4% of patients present with spinal metastases at the time of lung cancer diagnosis (12, 13). Surgical intervention has been shown to effectively control pain, restore spinal instability, preserve or restore neurological function, and improve patients’ quality of life (11, 14–16), complete resection can even be achieved in patients with oligometastases (17). However, some scholars argue that the efficacy of conservative treatment is comparable to that of surgical intervention (18). Thus, the optimal management strategy for lung cancer patients with spinal metastases remains controversial.

The objectives of this study are to evaluate the efficacy of surgical treatment in lung cancer patients with spinal metastases, identify the prognostic factors influencing postoperative 1-year survival, and provide evidence-based references for the surgical management of spinal metastatic tumors.

## 2 Patients and methods

### 2.1 Study subjects

This was a retrospectively study of inpatients with spinal metastases from lung cancer who received surgical treatment in Shenzhen People's Hospital from January 2017 to December 2022. The study has been approved by the Medical Research Ethics Committee of Shenzhen People's Hospital (LL-KY-2023136-01). All clinical and follow-up data of patients with lung cancer spinal metastases were collected.

**Inclusion criteria:**

1. Spinal metastatic tumor from lung cancer was identified by pathological diagnosis;
2. Age: more than 18 years of age;
3. Expected survival period more than 3 months;
4. Surgical indications: patients with refractory and intractable pain, progressive neurological compression or presence of spinal instability;
5. No serious diseases of the heart and brain and other important organs and systemic conditions to tolerate surgery;
6. Follow-up last for more than 1 year with complete data;

**Exclusion criteria:**

1. Patients with other types of spinal metastases
2. Patients with serious diseases such as severe cardiovascular and cerebrovascular diseases;
3. Patients died from other reasons or the follow-up data was incomplete;
4. Patients without surgery were excluded.
5. Patients or their family members refuse surgical treatment

According to the inclusion criteria and exclusion criteria, a total of 55 patients were included.

### 2.2 Surgical procedure

The surgical plan of each patient was discussed in the department of spinal surgery. Surgical interventions consisted of curettage surgery or percutaneous vertebroplasty or a combination of both. (1) Curettage surgery: tumor lesion curettage can remove the lesions in the vertebra, effectively expand the volume of the spinal canal, and improve the symptoms of pain and nerve compression in patients, It is suitable for patients with acute and progressive neurological impairment, patients with poor spinal stability, severe pain, or neurological dysfunction. (2) Percutaneous vertebroplasty: Patients suffered from spinal metastatic pain caused by symptomatic or stable vertebral compression fracture without neurological impairment. The surgery can effectively relieve pain, restore the height of the compressed vertebral body, improve spinal stability, and prevent further vertebral collapse. 11 cases underwent curettage and decompression with or without internal fixation, and 34 cases underwent percutaneous vertebroplasty. Curettage and decompression plus percutaneous vertebroplasty were performed in 24 cases.

### 2.3 Data Collection

Patients who underwent surgery at our institution was retrospectively reviewed at the end of April 1, 2024. Age, gender, height, weight, Body Mass Index (BMI), length of hospital stay, primary tumor type, metastatic stage, type of bone destruction, preoperative and postoperative adjuvant treatment plan (chemotherapy, radiotherapy, targeted therapy, immunotherapy), use of bone modifying agents (BMAS) were counted for all patients, presence of extra-spinal bone metastasis, presence of visceral metastases, presence of pathological vertebral fracture, postoperative complications (cerebrospinal fluid leakage/infection/hematoma formation/wound effusion/hemorrhagic shock), operation duration, intraoperative blood loss, postoperative drainage volume, preoperative and postoperative visual pain score (VAS), Frankel scale before and after surgery, Oswestry Disability Index (ODI score), survival time. VAS, ODI score and Frankel scale were used to evaluate the pain degree, functional status and neurological function of the patients respectively.

The date of surgery was used to be as the beginning of survival time. And if the patient underwent multiple surgeries, the date of the first surgery was taken. Tumor-specific death was recognized as the end event. This study set a final follow-up date of April 1, 2024. Follow up was employed through inpatient record system, outpatient visit or telephone. Imaging data including x-ray, CT, MRI and PET-CT of the primary and metastatic lesions of lung cancer were collected. Tumor progression: New metastases, or progression or recurrence of the primary lesion were thought as tumor progression by anyone of the imaging data B-ultrasound, x-ray, CT, MRI, or PET-CT after surgery.

## 3 Statistical analysis

All data were analyzed using SPSS version 27.0 software. The data conforming to normal distribution were expressed as mean ± standard deviation, while the non-normal distribution data were expressed as median (lower quartile to upper quartile). T test or non-parametric test was used for quantitative data difference test, and Chi-square test was used for qualitative data difference test. Kaplan–Meier method was used to draw the survival curve and Log-rank test was used for single factor analysis. Multivariate Cox proportional risk model was used to analyze the independent factors related to one-year survival. *P* < 0.05 was considered statistically significant.

## 4 Results

### 4.1 Patient characteristics

In total, 55 lung cancer patients with spinal metastases were identified, including 45 lung adenocarcinoma (81.82%), 4 lung squamous cell carcinoma (7.27%), 5 lung small cell carcinoma (9.09%), and 1 lung lymphoepitheliomatoid carcinoma (1.82%). There were 29 males (52.73%) and 26 females (47.27%). The mean age was 57.36 ± 10.38 years old, 58.86 ± 10.19 years old for males and 56.00 ± 10.54 years old for females. Of these 55 patients 37 were osteolytic changes (67.27%), 13 were osteoblastic changes (23.64%) and 5 mixed changes (9.09%).

8 cases (14.55%) underwent curettage and decompression internal fixation, 29 cases (52.73%) underwent percutaneous vertebroplasty, and 18 cases (32.73%) underwent curettage and decompression internal fixation plus percutaneous vertebroplasty. External spinal bone metastasis occurred in 36 cases (65.45%). There were 36 cases (65.45%) with visceral metastases. Vertebral body pathological fracture in 29 cases (52.73%); Preoperative chemotherapy in 21 cases (38.18%); Preoperative radiotherapy in 8 cases (14.55%); Preoperative targeted therapy in 26 cases (47.27%); Preoperative immunotherapy was performed in 5 cases (9.09%); Preoperative Bone modifying agents (BMA) were used in 14 cases (25.45%). Postoperative chemotherapy in 25 cases (45.45%); Postoperative radiotherapy in 13 cases (23.64%); Postoperative targeted therapy in 45 cases (81.82%); Postoperative immunotherapy was performed in 9 cases (16.36%). Postoperative BMA were used in 30 cases (54.55%). Emergency operation was performed in 3 cases (5.45%); There were 23 patients (41.82%) with abnormal neurological function before surgery and 17 patients (30.91%) with abnormal neurological function after surgery. Follow-up showed that neurological function of 36 cases (65.45%) was progressed. Postoperative complications occurred in 9 cases (16.36%), including hemorrhagic shock in 1 case, postoperative infection in 5 cases (pulmonary infection in 4 cases, wound infection in 1 case), cerebrospinal fluid leakage in 1 case, hematoma in 2 cases. The operation duration was 150.00(85.00,279.00) min, the amount of blood loss during the operation was 30.00(6.50,800.00) mL, the postoperative drainage was 0.00(0.00,610.00) mL, and the length of hospitalization was 15.00(9.00,20.00) days.

### 4.2 Comparison of pain and functional status before and after surgery

According to Table 1: VAS score before surgery was 5.00 (3.00, 7.00), and the VAS scores 1 month, 3 months, and 6 months after surgery were statistically significant compared with the VAS score before surgery (*P* < 0.05), and all of them were lower than the VAS score before surgery. The ODI score before surgery was 57.50 (25.00, 82.50), and the ODI scores 1 month, 3 months, 6 months, and last visit were statistically significant compared with the ODI score before surgery (*P* < 0.05), and all of them were lower than that before surgery, while the last ODI score was higher than that before surgery.

**Table 1:** Comparison of VAS score and ODI score of patients before and after surgical treatment [M (P25, P75)].

Time	VAS	ODI
Before surgery	5.00 (3.00, 7.00)	57.50 (25.00,82.50)
1 month after operation	1.00 (0.00, 3.00)^a^	37.50 (15.00,53.75)^a^
3 month after operation	1.00 (0.00, 3.00)^a^	22.50 (10.00,54.46)^a^
6 month after operation	2.00 (0.00, 3.00)^a^	15.00 (5.00,43.13)^a^
Last time	5.00 (2.00, 7.00)^b^	84.44 (15.00,93.33)^a^

a Compared with the preoperative group, *p <* 0.05.

b Compared with the preoperative group, *p >* 0.05.

### 4.3 Comparison of neurological function before and after surgery

Frankel score was used to analyze the neurological functionas. The chi-square test was used to compare the Frankel scores before and after surgery. See Tables 2, 3.

**Table 2:** Frankel grading of patients before and after surgery.

Before surgery	Number	After surgery				
		A	B	C	D	E
B	7	0	1	1	3	2
C	4	0	0	0	3	1
D	12	0	0	0	7	5
E	32	1	1	0	1	30
Total	55	1	2	1	13	38

**Table 3:** Frankel grading of patients before and after surgery.

Matching data	Name	Frankel grading after surgery		Total	*χ^2^*	*p*
		abnormal	normal			
Frankel grading before surgery	abnormal	15	8	23	21.79	0.000**
	normal	2	30	32		
Total		17	38	55		

**p < 0.01.

There was a statistically significant difference in the improvement of Frankel grade in neurological function before and after surgery (*χ*^2^ = 21.79, *P* = 0.000). Of the 23 patients with neurological dysfunction before surgery (Frankel grade of B to D), 32 patients had normal neurological function before surgery (Frankel grade of E). Of the 23 patients with neurological dysfunction before surgery, 8 recovered normal neurological function after surgery, 8 maintained their neurological function, 7 did not recover normal neurological function but improved compared to before surgery, and 2 had worsened neurological function after surgery. One patient developed paraplegia one month after surgery, which was considered to be caused by tumor progression after imaging examination. One patient's neurological function had recovered to normal one year after follow-up.

### 4.4 Survival analysis

The median survival time of 55 patients in this study was 16.83 months, 95%CI (9.88, 23.78), and one-year survival rate was 58.18%. Kaplan–Meier survival curve and Log-rank test were used to analyze the factors affecting the one-year survival rate of patients with lung cancer spinal metastasis. According to Tables 4, 5: Age, sex, BMI, length of hospital stay, preoperative VAS score, postoperative VAS score (1 day), postoperative VAS score (3 days), postoperative VAS score (7 days), postoperative VAS score (3 months), postoperative VAS score (6 months), postoperative VAS score (last time), Preoperative ODI score, postoperative ODI score (3 months), postoperative ODI score (6 months), postoperative ODI score (last time), operation duration, blood loss mL, total drainage volume mL, tumor type, operation type, multi-stage (≥3) metastasis, type of bone destruction, whether there was extra-spinal bone metastasis, whether there was pathological vertebral fracture, preoperative chemotherapy, preoperative radiotherapy, and operation Preoperative endocrine therapy, preoperative targeted therapy, preoperative immunotherapy, preoperative bone modification agent, postoperative chemotherapy, postoperative radiotherapy, postoperative endocrine therapy, postoperative targeted therapy, postoperative immunotherapy, preoperative Frankel scale, postoperative Frankel scale, emergency surgery, there were no significant differences in the effects of complications or progression on the postoperative 1-year survival (*P* > 0.05). ODI score (1 month) (Z = −2.353, *P* = 0.019), whether there was visceral metastases (*χ*^2^ = 5.145, *P* = 0.023), whether bone modifying agent (BMA) were used after surgery (*χ*^2^ = 6.227, *P* = 0.013) had significant differences on the postoperative 1-year survival of patients (*P* < 0.05).

**Table 4:** The results of univariate analysis of survival risk factors in surgical patients [M (P25, P75)].

Factors	Prognosis		*Z*	*P*
	Survival (*n* = 32)	Mortality (*n* = 23)		
Postoperative ODI score (1 months)	30.00 (10.63, 48.75)	47.50 (26.25, 66.25)	−2.353	0.019*

*p < 0.05.

**Table 5:** The results of univariate analysis of risk factors for survival in surgical patients.

Factors		Prognosis (%)		*χ^2^*	*P*
		Survival	Mortality		
Visceral metastases	No	15 (46.88)	4 (17.39)	5.145	0.023*
	Yes	17 (53.13)	19 (82.61)		
Postoperative bone modifying agent (BMA)	No	10 (31.25)	15 (65.22)	6.227	0.013*
	Yes	22 (68.75)	8 (34.78)		

*p < 0.05.

### 4.5 Multi-factor comprehensive analysis

**Figure 1:**
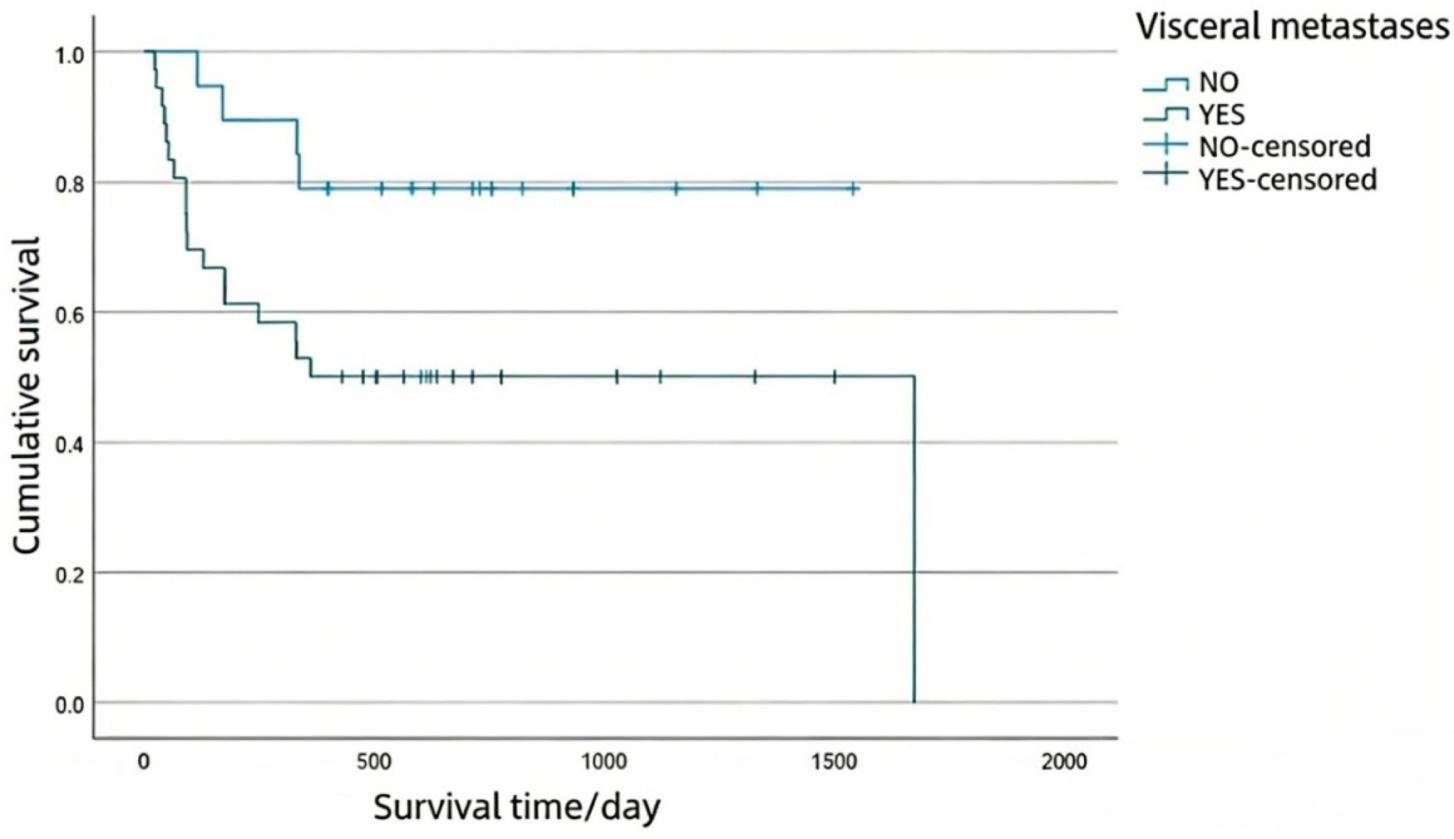
Overall survival (OS) of patients with or without visceral metastases.

**Figure 2:**
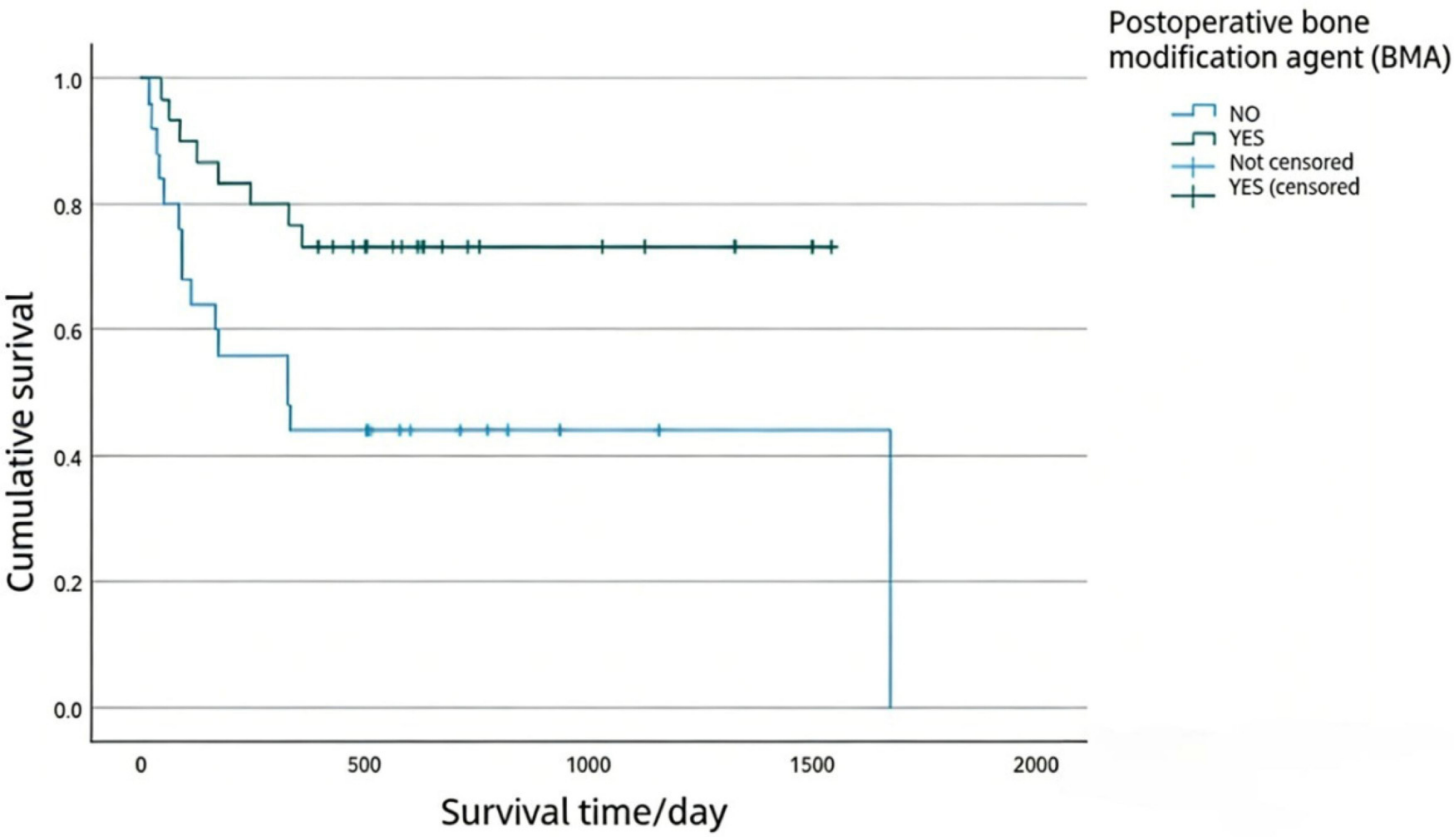
Overall survival (OS) of patients treated with bone modifying agents (BMA) postoperatively.

The above factors were incorporated into the Cox proportional risk model for multivariate analysis, and the results showed that visceral metastases and postoperative bone modifying agent (BMA) were independent factors affecting the postoperative 1-year survival of patients (Table 6). Kaplan-Meier survival curves were generated for patients with spinal metastases from lung cancer, stratified by the presence of visceral metastases and the administration of bone-modifying agent (BMA) after surgery (Figures 1, 2).

**Table 6:** Multivariable Cox proportional hazards model for survival analysis.

Factors	*B*	*P*	*Exp (B)*	95.0%CI
visceral metastases	1.263	0.023	3.538	1.191 ± 10.516
Postoperative bone modifying agent (BMA)	−1.118	0.012	0.327	0.136 ± 0.784

## 5 Discussions

According to the report of the World Health Organization, lung cancer is the most common malignant tumor and is also considered to have the worst prognosis because of the shortest median survival time (1, 3, 11). With the development of multimodal treatment for lung cancer, the 5-year survival rate of lung cancer in China has also increased from 16.1% (2003–2005) to 19.7% (2012–2015) (19), and the survival period of lung cancer patients has been significantly extended, which also leads to an increased probability of tumor-related bone events (2). The spine is the most common part of bone metastasis, and spinal metastasis is very common in patients with advanced malignant tumors (3–6), resulting in spinal cord compression, pathological fracture, severe pain, paralysis and other symptoms, which seriously affect the life and quality of life of patients (8, 10). The purpose of surgical treatment for spinal metastatic tumor is to relieve compression, restore or maintain nerve function, restore spinal stability, relieve pain related to spinal tumor metastasis, and correct spinal deformity, so as to improve patients’ quality of life (20). In this study, VAS score (5.06 ± 2.15) and ODI score (52.38 ± 30.45) before surgery, VAS score at 1 month, 3 months and 6 months after surgery, ODI score at 1 month, 3 months and 6 months after surgery were all decreased compared with that before surgery, and patients’ pain and living ability were significantly improved. There was no statistical difference between the last VAS score after surgery and the VAS score before surgery, and the last ODI score was statistically different from the preoperative ODI score, which was significantly higher than the preoperative ODI score, considering the tumor progression over time. In terms of the type of surgery, there was no statistically significant difference in pain relief between tumor curettage and decompression internal fixation, percutaneous vertebroplasty, tumor curettage and decompression internal fixation plus percutaneous vertebroplasty.

In terms of neurological function, the Frankel rating of the patients was significantly improved after surgical treatment, and the neurological function of 8 of the 23 patients with abnormal neurological function before surgery returned to normal after surgery. The neurological function remained unchanged in 8 cases and did not return to normal in 7 cases, but it was improved compared with before operation, and the improvement rate of neurological function was 65.22%. By comparing the VAS score, ODI score and Frankel scale of patients before and after surgery, it was confirmed that surgical treatment can effectively improve the pain of patients, improve the quality of life of patients, and restore or maintain nerve function. Chan et al. (21) performed surgery on 49 patients with spinal metastases and studied the effects of surgery on survival and nerve function, and the results showed that surgery could improve or maintain nerve function for a long time. –Shehadi et al (22) conducted a study on 133 patients with metastatic tumors who received surgical treatment, among which 46.7% of the patients with abnormal neurological function before surgery recovered to normal after surgery, indicating that active surgical management can significantly relieve pain and maintain or improve nerve function. A multi-center study by Yan et al. (23) divided 343 patients with patients with spinal metastases from lung cancer into an operation group and compared them with a non-operation group, and concluded that surgical treatment can significantly improve the neurological function and general status of patients with spinal metastases from lung cancer. The above studies are basically consistent with the results of this study, and surgery is effective in the treatment of lung cancer spinal metastases.

Lei and Miao (24) believed that the presence or absence of visceral metastases and multiple spinal metastasis in spinal metastases were related factors affecting the prognosis of patients. A retrospective analysis of 577 cases of spinal metastases by Lee et al. (25) showed that clinical symptoms, visceral metastases, and pathological type of primary tumor were related to survival. Univariate survival analysis in this study showed that patients’ ability to live, presence or absence of visceral metastases, and use of postoperative bone modifying agent (BMA) were influencing factors for patients’ postoperative 1-year survival. The above factors were included in Cox proportional risk model for multivariate analysis, and the results showed that the presence of visceral metastases and postoperative bone modifying agent (BMA) were independent factors affecting the postoperative 1-year survival of patients. Bone metastasis of lung cancer destroys the balance between bone resorption and bone formation, resulting in bone injury. Zoledronic acid can inhibit the activity of osteoclasts, thereby reducing the ability of osteoclasts to destroy bone tissue, maintaining the current bone mass and reducing the incidence of bone-related events, which has become the basic means of bone metastasis treatment. Studies have shown that chemotherapy and radiotherapy combined with zoledronic acid have significant clinical efficacy in the treatment of lung cancer bone metastasis, which can effectively improve bone pain, reduce serum alkaline phosphatase and calcium ion levels, improve quality of life and survival rate, and drug tolerance and side effects are controllable. Uei et al. (26) conducted a retrospective analysis of 270 patients with lung cancer spinal metastasis and found that after the treatment of lung cancer metastatic spinal tumor with targeted therapy and bone modifying agent (BMA), the survival period of patients was prolonged and the ability of daily living was improved after treatment. In this study, the median survival time of lung cancer patients with spinal metastasis was 16.83 months, which was higher than the survival time reported in previous literature. This may be related to the strict grasp of surgical indications and the exclusion of some patients with short survival and standard treatment of patients with lung cancer spinal metastasis. In this study, only 4 patients did not receive systemic therapy after surgery, and systemic therapy significantly extended the survival time of patients. In addition, the genetic subtypes of tumors of the same case type also affect the prognosis of patients. Targeted therapy has shown superior efficacy in EGFR mutation-positive lung cancer patients, and the average survival of patients has been significantly extended (27, 28). Therefore, it is incorrect to simply classify all types of lung cancer as the tumors with the worst prognosis.

The study results of Li et al. (29) showed that targeted therapy and chemoradiotherapy are independent factors affecting the survival of lung cancer spinal metastases. Yan et al. (23) reported that radiotherapy, chemotherapy and targeted therapy can improve the one-year survival rate of patients with lung cancer spinal metastases. In this study, systemic therapy (chemotherapy, radiotherapy, targeted therapy, immunotherapy) had no significant effect on the postoperative 1-year survival of patients with lung cancer spinal metastases, but the mean survival time of patients receiving systemic therapy was higher than that of patients without systemic therapy. This may be related to the small sample size and patient bias.

The decision-making process of spinal metastatic tumor treatment is complex, with many factors affecting survival, difficult to predict expected survival, difficult to quantify preoperative factors and interdependent. In summary, surgical treatment can help patients relieve pain, maintain or improve nerve function, and improve quality of life. There are currently no widely accepted tools to accurately predict surgical outcomes based on the overall condition of patients with spinal metastatic disease, so in addition to considering prognostic scores, surgeons need to consider a variety of factors, such as tumor genetic subtypes, internal metastasis, and nutritional status. In determining the feasibility of surgical options, evaluation must be carried out according to the principle of individualization. Through multidisciplinary cooperation, individual treatment plans are developed, systemic therapy and bone modifying agent are standardized to prevent bone-related events, and patients’ conditions are dealt with by precise treatment, so that patients can obtain greater treatment benefits.

Notably, this study has inherent limitations that should be considered when interpreting the findings. The single-center retrospective design and the inclusion criterion of anticipated survival >3 months inevitably introduce selection bias, rendering the cohort potentially unrepresentative of the broader population of patients with spinal metastases from lung cancer and overrepresentative of those with favorable physical status and prognosis. Therefore, caution is warranted when generalizing the study conclusions to all such patients.

Furthermore, the retrospective design imposes additional limitations: data collected through multiple channels (case filing system, outpatient records, telephone follow-ups) may introduce biases in completeness and accuracy; the COVID-19 pandemic impeded regular follow-ups and standardized imaging re-evaluations, compromising the integrity of longitudinal data; and the small sample size precluded in-depth subgroup analyses. Specifically, the lack of systematic documentation of molecular testing data (e.g., EGFR, ALK, ROS1 gene status) in some enrolled cases prevented stratified analyses by tumor genetic subtypes—a critical limitation given the marked biological heterogeneity of lung cancer and the profound impact of molecular subtypes on survival prognosis and response to systemic therapies (e.g., EGFR TKIs, immunotherapy). Additionally, subgroup analyses based on laboratory biomarkers and comorbidities were not feasible, and the employment of subjective questionnaires may have introduced additional selection bias. Moreover, due to the retrospective nature of this study, data related to preoperative evaluation scores (e.g., Bauer score, NESMS score) were not fully recorded in the clinical data of some early enrolled cases, making systematic analysis of these scores impossible. This is another notable limitation, as such preoperative scores are important for assessing the prognosis of patients with spinal metastases and verifying the rationality of surgical intervention.

Notably, our finding of an independent association between postoperative bone modifying agent (BMA) use and improved survival, while clinically relevant, is susceptible to confounding by unmeasured variables. Although common clinical covariates (e.g., age, gender, tumor stage, performance status, presence of visceral metastases, receipt of systemic therapy) were adjusted for in multivariate analyses, unquantifiable factors—such as disparities in access to systemic care and overall management quality, which may be superior in BMA recipients—could not be fully accounted for. Thus, this association should be interpreted with caution: BMA-related survival benefits are likely mediated indirectly via inhibition of skeletal-related events, pain relief, and improved quality of life, rather than through direct oncological effects.

Despite these limitations, this study provides valuable clinical insights: surgical intervention effectively alleviates pain, preserves or improves neurological function, and enhances quality of life in patients with spinal metastases from lung cancer. Visceral metastases and bone modifying agents (BMA) postoperatively are independent prognostic factors for 1-year postoperative survival. These findings underscore the clinical utility of surgical treatment for this challenging patient population, despite the technical complexity of spinal metastasectomy.

Future multi-center, large-sample prospective studies are required to validate and extend these conclusions, as such designs will minimize selection bias and confounding factors, thereby enhancing external validity and generalizability. Additionally, future research should enroll patients with complete molecular testing data to conduct comprehensive subgroup analyses based on tumor biology, laboratory biomarkers, and comorbidities. Prospective designs are also needed to further verify the survival value of BMA through rigorous matching or adjustment for relevant confounding factors, ultimately facilitating the development of personalized treatment strategies for patients with spinal metastases from lung cancer.

## Data Availability

The original contributions presented in the study are included in the article/Supplementary Material, further inquiries can be directed to the corresponding authors.
